# Supplementary material for: Is vaccination a viable method to control Johne’s disease caused by Mycobacterium avium subsp. paratuberculosis? Data from 12 million ovine vaccinations and 7.6 million carcass examinations in New South Wales, Australia from 1999–2009
Source: PLoS One. 2021 Jun 14;16(6):e0246411. doi: 10.1371/journal.pone.0246411 (PMC8202914; doi:10.1371/journal.pone.0246411)
Supplement: Attachment — Submitted filename: PONE-D-21-01240 response.docx [file pone.0246411.s002.docx]

## PLOS1 OJD Vaccination & Abattoir Monitoring Cover Letter - 040121

## To the Editor, PLOS1

On behalf of my co-authors, I thank you for consideration of our manuscript for publication in PLOS1.

**“Is Vaccination a Viable Method to Control Johne’s Disease Caused by *Mycobacterium avium* subsp. *paratuberculosis*? Data from 12 Million Ovine Vaccinations and 7.6 Million Carcass Examinations in New South Wales, Australia from 1999-2009”**

Please find below relevant supporting information.

Yours Faithfully

**Ian J Links,**

**Graham Centre for Agricultural Innovation, Wagga Wagga NSW 2650**

**+61 457643292,**

**ijlinks48@gmail.com.**

# Contribution to the scientific literature - Background

*Mycobacterium avium* subsp. *paratuberculosis* (MAP) causes Johne’s disease (or paratuberculosis), a chronic wasting disease of ruminants and other animals resulting from granulomatous enteritis. There are increasing concerns that MAP is zoonotic. The prevalence of Johne’s disease is increasing worldwide. In an attempt to control an epidemic of ovine Johne’s disease (OJD) in New South Wales (NSW), a government/industry sponsored voluntary vaccination/on-farm management program commenced in 2000.

We document an observational study of changes in disease prevalence as vaccination progressed, based on abattoir surveillance data for OJD from 1999 to 2009. We also discuss the epidemiological, policy, regulatory, research, economic and sociological elements that contributed to the development of a mature control program, whose aim was to halt the exponential spread of OJD in a naïve sheep population.

Development of abattoir monitoring with inspection of viscera followed by confirmatory histopathology proved a major breakthrough for surveillance by enabling cost effective detection of sub-clinical infection on a state wide basis. Feedback to producers (dependent on fast-tracking implementation of a National Livestock Identification System - NLIS) encouraged vaccination in infected flocks (Gudair® heat killed oil adjuvant) with progressive build-up of flock and regional “herd immunity”. Combined with evolving knowledge of management procedures in minimising faeco-oral exposure of susceptible animals, these two core approaches were instrumental in helping breaking the cycle of infection.

When the study commenced there was little evidence to suggest that either abattoir monitoring or vaccination would be effective in achieving the desired goals. Historical reports of the efficacy of killed vaccines from Iceland and Spain were encouraging. With many flocks in NSW reporting ≥5% annual mortality in adult sheep, ‘Gudair®” was approved for emergency use in early 2000 but restricted to newborn lambs (4-16 weeks of age) nominally prior to exposure to MAP. Subsequently, in mid-2000, preliminary results confirmed that it was safe to vaccinate adult sheep even in a heavily infected flock with an estimated 19% annual mortality [1]. Nonetheless, widespread vaccination was not approved until 2002 following demonstration of a 90% reduction in both clinical disease and bacterial excretion following vaccination of lambs in 3 heavily infected flocks [2].

Abattoir monitoring, implemented in late-1999, rapidly proved sensitive, specific and cost effective in detecting sub-clinical infection. It rapidly replaced on-farm surveillance testing. Feedback to producers drove uptake of vaccine while providing comprehensive state-wide surveillance data, enabling clearer definition of prevalence areas.

The control program met considerable producer resistance (in heavily affected areas) due to the economic impact of quarantine on producers and their community. In contrast, producers with flocks in areas with low prevalence were strongly supportive of increased protection. Understanding and dealing with the socio-economic aspects of the disease and control measures were critical in moving from a traditional top-down regulatory approach based on Quarantine to a novel Risk-based producer driven program akin to that implemented in the Human AIDS pandemic in the 1990’s.

This report documents the marked reduction in prevalence of sub-clinical disease (as assessed by abattoir monitoring of adult sheep) in the NSW high prevalence area (from 2.4% of sheep monitored in 2000 to 0.8% in 2009) in parallel with widespread use of vaccine (estimated 70% of adult flock vaccinated by 2009).

The potential therapeutic benefit of the killed whole cell vaccine, in already infected sheep, remains a source of uncertainty among researchers – a critical question requiring further investigation.

This report documents, from 1999-2009, a great success story – effective control of a mycobacterial disease (long-incubation period, intracellular infection) that threatened the future of the Australian sheep industry. Concurrently there was a dramatic reduction in reported cases of clinical disease and environmental contamination. It required the dedicated efforts of very large numbers of professional and lay personal from Industry and Government over more than a decade to achieve this outcome.

Abattoir monitoring for OJD continues to this day in sheep abattoirs across Australia with feed-back to producers promoting vaccination. Since 2007 a wide range of “Other Conditions” visible at slaughter have been reported to producers utilising the same inspection and reporting system as for OJD. These include such diverse conditions as OJD vaccine abscesses, dog bites, liver fluke, pneumonia, broken ribs, hydatids etc. and comes at no additional cost.

There is an ongoing international pandemic of Paratuberculosis in cattle (particularly dairy cattle) [3] which is being ineffectively managed. The existing ineffective test-and-cull/management approach should be complemented and then replaced by a new approach incorporating vaccination (currently resisted due to concerns of interference with Tuberculosis testing) and abattoir monitoring.

## Relationship to previously published work

The elements of the control program are discussed in the context of previously published work.

Type of article

“Observational Study of an Epidemic Disease Control Program”

Prior interactions with PLOS regarding the submitted manuscript

Prior submission in September 2016 - PONE D-16-36925. Reviewers recommended acceptance subject to major revision. The submission was withdrawn after 6 months.

## Response to Major Issues Raised by Editor/Reviewers (September 2016)

1. The 2 maps (Figs. 1 & 2) are subject to NSW DPI copyright – Formal Creative Commons approval for use of maps has now been provided by Dr Jim Rothwell, Director, EMAI, NSW Central Veterinary Research Laboratory, NSW DPI Menangle, NSW (approval uploaded). The lead author (IJL) supplied the data and oversaw the construction of both these maps including defining the position of prevalence area boundaries based on location of known positive flocks.
2. The Tables have not been consolidated. Because of the extended timeframe of the study and the data for multiple prevalence areas, this would have made the review of the data more complex for the reader.
3. The paper has been rewritten to provide a more cohesive coverage of the interplay of the elements critical to the success of the evolving control program. Sub-headings have been added for easier reading. Undoubtedly there were many elements that contributed to the successful control of OJD in NSW and it is important to recognise their role in achieving a successful outcome. This has meant an increase in the length of the manuscript but we consider this warranted in terms of the importance of the subject matter to the international and Australian authorities responsible for management of epidemic diseases in general, and Johne’s disease in particular.
4. Some sections have been covered in more detail in the Supplementary Text. A list of these Supplementary sub-headings has been added at the end of the manuscript. They are numbered for easy cross-reference.
5. The references to Conference Proceedings, while not formally Peer Reviewed and in many cases presented by the current Authors, have been retained. Prior to acceptance, they were all subjected to review by an editorial committee with international expertise in Johne’s Disease. It is critical in the face of a pandemic/epidemic to have data and findings published in a timely fashion to ensure rapid dissemination of information. Please note that over the decade covered by this paper we were working in a complex disease control response dealing directly with the rural community, National veterinary authorities and the sheep industry, not in a research academic environment.
6. We have elected to re-write the paper as an “observational study”. While it may be possible to subject the data to detailed statistical analysis, we consider that the large numbers in the data set, combined with the standardised approach to data collection and interpretation, provide strong evidence for the conclusions reached.
7. Figures have been added showing typical histopathological changes due to OJD affecting the terminal ileum - Multibacillary OJD lesions with masses of acid-fast organisms (AFOs) present and Paucibacillary lesions with AFOs absent.
8. A figure showing location of sampling sites has also been added.
9. A flow diagram suggested by one reviewer has not been included as it was considered likely too complicated and confusing.
10. Reference to the potential zoonotic importance of Paratuberculosis in Crohn’s Disease has been retained as has the comparison with bovine Paratuberculosis due to the importance of these issues from a OneHealth perspective.
11. A list of Abbreviations has been added at the end of the manuscript
12. The underpinning baseline data from this project (with producer’s identity masked) is available at:

[**http://www.dpi.nsw.gov.au/__data/assets/file/0011/675731/PLOS1-Links-Raw-Abattoir-Data-1999-2009.zip**](http://www.dpi.nsw.gov.au/__data/assets/file/0011/675731/PLOS1-Links-Raw-Abattoir-Data-1999-2009.zip)

## **Suggested Academic Editor**

Prof. [Michael T. Collins,](mailto:michael.t.collins@wisc.edu) DVM, PhD, DACVM

Professor Emeritus, University of Wisconsin-Madison

University of Wisconsin School of Veterinary Medicine | 2015 Linden Drive, Madison, WI 53706-1102

Email: [Michael.T.Collins@wisc.edu](mailto:Michael.T.Collins@wisc.edu) or mcollin5@wisc.edu

Prof. Collins is widely recognised as an international expert on Paratuberculosis (Johne’s Disease).

There are no “Opposed Reviewers”.

**References**

1.	Windsor P. A longitudinal study of OJD and the effects of whole flock vaccination with Gudair - Whole flock OJD vaccination at Merrill - OJD.015. Technical Report. North Sydney, NSW, Australia: University of Sydney, 2005 March 2005. Report No.

2.	Reddacliff L, Eppleston J, Windsor P, Whittington R, Jones S. Efficacy of a killed vaccine for the control of paratuberculosis in Australian sheep flocks. Veterinary microbiology. 2006;115(1-3):77-90. PubMed PMID: 16459030.

3.	Whittington R, Donat K, Weber MF, Kelton D, Saxmose Nielsen S, al. e. Control of paratuberculosis: who, why and how. A review of 48 countries. BMC Veterinary Research. 2019;15:29.
