# Supplementary material for: Is vaccination a viable method to control Johne’s disease caused by Mycobacterium avium subsp. paratuberculosis? Data from 12 million ovine vaccinations and 7.6 million carcass examinations in New South Wales, Australia from 1999–2009
Source: PLoS One. 2021 Jun 14;16(6):e0246411. doi: 10.1371/journal.pone.0246411 (PMC8202914; doi:10.1371/journal.pone.0246411)
Supplement: Attachment — Submitted filename: PLOS1 Reviewers Authors Response V3AB 7-13pm 090321.docx [file pone.0246411.s003.docx]

9 March 2021

Dr. Yung-Fu Chang
Academic Editor
PLOS ONE

Dear Dr. Yung-Fu Chang:

**re: Manuscript PONE-D-21-01240**

Please find a revised version of our manuscript " Is Vaccination a Viable Method to Control Johne’s Disease Caused by *Mycobacterium avium* subsp. *paratuberculosis*? Data from 12 Million Ovine Vaccinations and 7.6 Million Carcass Examinations in New South Wales, Australia from 1999-2009" for reconsideration for publication in PLOS One. In preparing this revised version of our manuscript, we would like to thank the two reviewers for their thorough review and helpful comments in order to improve the overall quality of the report.

Below are our responses to the editor’s requests and the comments of the two reviewers.

## Editor

**Comment 1**: The editor requested that the manuscript meets PLOS ONE's style requirements, including those for file naming.

**Response:** The manuscript has been revised to meet the journal’s style requirements.

Comment 2: The editor requested that the authors included the names of the slaughterhouses where the sheep were humanely killed.

Response: The names of the abattoirs have been included in the revised manuscript (see subsection “Abattoir monitoring” page 13, lines 238-242).

Comment 3: The editor requested that the figure meet PLoS requirements using the PACE digital diagnostic tool.

Response: The figure files have been converted to TIF format and have been checked on PACE before upload.

## Reviewer 1

Comment 1: The reviewer commented that the authors succeeded in developing an informative review highlighting the events that led to successful control of paratuberculosis in sheep using a killed vaccine.

Response: The authors thank the reviewer for the compliments on the quality of the informative review and the clarity, concision, and coherence of the report.

## Reviewer 2

Comment 1: The reviewer commented that some parts of the manuscript’s text were not reader-friendly, contained excessive details, and some information in the Methods and Discussion sections could be incorporated into “Supplementary Text”.

Response: We critically reviewed the manuscript’s text to determine whether we could comply with the reviewer’s request to improve the manuscript’s readability and still convey an informative message with enough details (see comments of reviewer 1). Longer paragraphs have been split to improve readability.

The following sections have been retained: Vaccination Procedures; Vaccination discussion; Property Disease Management Plans (PDMPs) & Financial Assistance; Sheep Breeds.

The following sections have been shortened by transfer to Supplementary Text: Feedback to Producers (S6); Susceptibility of Sheep Breeds to Infection (S7a).

Comment 2: The reviewer suggested the authors might consider moving table 1 and table 3 to supplementary data.

Response: Tables 1 and 3 have been retained in the manuscript. Consignments were the primary unit monitored with flock-level prevalence a critical parameter of the study.

Comment 3: Is MAP 316F the dominant strain in Australia sheep suffering from OJD?

Response: No. For all practical purposes there is only one dominant Sheep S1 strain in Australia [1] for which Gudair vaccine containing MAP 316F (a bovine vaccine reference strain) provides excellent protection. The Telford strain, for which there is a whole genome sequence [2], is representative of the endemic S strain in Australia and New Zealand. This background details with references have been added to the Introduction (Lines 63-66, P5)

Comment 4: The authors may use statistical analysis to elucidate the correlation among OJD, vaccine doses, and herd immunity.

Response: While statistical analysis may be possible it has not been pursued at this stage. In essence, the large data set for animals monitored and vaccinated should provide strong statistical support for the validity of the conclusions reported. We are hopeful that publication of the manuscript will stimulate offers to undertake statistical analysis – we would be happy to co-operate in such an undertaking.

If there are any additional concerns and/or comments about the revised manuscript, please do not hesitate to contact me.

We look forward to receiving the editorial decision in the very near future.

Yours sincerely,

Ian J. Links

Corresponding Author
